# Supplementary material for: Identification, Typing and Drug Resistance of Cronobacter spp. in Powdered Infant Formula and Processing Environment
Source: Foods. 2023 Mar 3;12(5):1084. doi: 10.3390/foods12051084 (PMC10000698; doi:10.3390/foods12051084)
Supplement: Supplementary file 1 [file foods-12-01084-s001.zip › foods-2160383-supplementary.pdf]

Supplementary Tables

Table S1. Bacterial strains used in this investigation and their sources

Strains	Bacterial species	Area	Source
CS1	*C. sakazakii*	Northeast	PIF
CS2	*C. sakazakii*	Central	PIF
CM3	*C. malonaticus*	Northern	PIF
CS4	*C. sakazakii*	Northeast	PIF
CS5	*C. sakazakii*	Northeast	PIF
CS6	*C. sakazakii*	Northeast	PIF
CS7	*C. sakazakii*	Northeast	End product of workshop
CS8	*C. sakazakii*	Northeast	PIF
CS9	*C. sakazakii*	Northeast	PIF
CS10	*C. sakazakii*	Northern	PIF
CS11	*C. sakazakii*	Northeast	PIF
CS12	*C. sakazakii*	Northern	PIF
CS13	*C. sakazakii*	Northern	PIF
CS14	*C. sakazakii*	Southern	PIF
CS15	*C. sakazakii*	Northeast	Whey powder
CM16	*C. malonaticus*	Northern	PIF
CS17	*C. sakazakii*	Northeast	Milk powder raw materials
CS18	*C. sakazakii*	Northeast	PIF
CS19	*C. sakazakii*	Northeast	PIF
CS20	*C. sakazakii*	Northern	PIF
CS21	*C. sakazakii*	Northern	PIF
CM22	*C. malonaticus*	Northeast	PIF
CT23	*C. turicensis*	Northern	PIF
CM24	*C. malonaticus*	Northeast	PIF
CS25	*C. sakazakii*	Northeast	PIF
CS26	*C. sakazakii*	Northeast	PIF
CS27	*C. sakazakii*	Northeast	Milk powder raw materials
CT28	*C. turicensis*	Northern	PIF
CM29	*C. malonaticus*	Northern	PIF
CS30	*C. sakazakii*	Northern	PIF
CD31	*C. dublinensis*	Northern	PIF
CS32	*C. sakazakii*	Northwest	PIF
CS33	*C. sakazakii*	Southern	Milk powder additive
CS81	*C. sakazakii*	Northeast	PIF
CS100	*C. sakazakii*	Northeast	End product of workshop

Table S2. 16S rRNA and 7 sets of PCR primers for housekeeping genes

Primers	Primer sequences (5'→3')
27-F	AGTCTCTGATCATGCCTCAG
1492-R	AAGGAGGTGCTCCAGCC
atpD-F	CGACATGAAAGGCGACATCGAAATGACCGACTCCAA
atpD-R	TTAAAGCCACGGATGGTGGGATGGCGATGATGTCTT
fusA-F	GAAACCGTATGGCGTCAGGCTGGATGCGGTAATTGA
fusA-R	AGAACCGAAGTGCAGACGCCCATACCAGCGATGATG
glnS-F	GCATCTACCCGATGTACGGGGTGCTGGATAACATCA
glnS-R	TTGGCACGCTGAACAGACCTTGTTGGCTTCTTCACG
gltB-F	CATCTCGACCATCGCTTCGCGAATACCACGCCTACA
gltB-R	CAGCACTTCCACCAGCTCGCGTATTTCACGGAGGAG
gyrB-F	TGCACCACATGGTATTCGCTCGCGGGTCACTGTAAA
gyrB-R	CACCGGTCACAAACTCGTACGCCGATACCGTCTTTT
infB-F	GAAGAAGCGGTAATGAGCTGACCACGGTAAAACCTC
infB-R	CGATACCACATTCCATGCGGACCACGACCTTTATCC
ppsA-F	GTCCAACAATGGCTCGTCACCCTGACGAATTCTACG
ppsA-R	CAGACTCAGCCAGGTTTGCAGATCCGGCATGGTATC

Note: F stands for forwarding sequence and R stands for reverse sequence

Table S3. Target genes and primers of qRT-PCR

Gene	Primer sequences (5'→3')
emrA (CSK29544_01824)	F: CGACTTTCACCAGCGTGGACAG
	R: TGGATCAAAGTGGTTCAGCGTCTG
CSK29544_03309	F: CGCCCTGCTCTATAACGACAACC
	R: GCCACTGCCGATCACCTGAATC
yojI (CSK29544_02362)	F: GACGCTCGCCATGCTCTTAACG
	R: AACAGCCAGACATCGGTGAACAC
mdfA (CSK29544_03801)	F: ATCTGCTCGCCGCTGATGATAATG
	R: CGTGAACTGGGCCGTGACTATAAAG
fliM (CSK29544_02582)	F: TCACTCATTGGGCTGTTCCTCATTC
	R: CTGGTGCATGTGGATGGCGTAC
mdlA (CSK29544_04123)	F: AAACCACTATCGCAATCACCACCAG
	R: CGGCGTCACTCAACACCACTAC
emrB (CSK29544_03823)	F: CTGGACGGCGGGTGTAGATGAG
	R: CGGCGAAGACGAAGACTGGTTC
pump (CSK29544_00631)	F: TGTTTGGGCGAATAATCTCCCTGAC
	R: CGCATACCACGCTTACGGACATC
CSK29544_03853	F: GCGACGGGATGTTAATTCTCTGGAG
	R: CGCCTTCGTTCATCAGCAGTATCTC
baeR (CSK29544_02460)	F: CGATTCTGGCGGTCACCATCAC
	R: GCGATCAGGTGTTGCCCTATGTG
16S rRNA	F: GGTGTAGCGGTGAAATGCGTAGAG
	R: CATCGTTTACGGCGTGGACTACC

Table S4 RNA quality test results of CS14

Sample Name	NanoDrop Concentration (ng/μL)	OD260/280	OD260/230	RIN
A1	1373.56	2.11	2.26	10
A2	1252.36	2.12	2.31	10
A3	1170.16	2.10	2.30	10
B1	1229.76	2.11	2.33	10
B2	1568.34	2.12	2.36	10
B3	1161.17	2.13	2.35	10

Note: The concentrations of all samples were higher than 1000 ng/μL, and OD260/280 and OD260/230 were both greater than 2.0, indicating that the concentrations and purity of the extracted RNA were extremely high. Meanwhile, the results showed that the RIN values of the samples were all 10 points, and the integrity index was qualified, indicating that the RNA was not degraded and met the requirements of cDNA library construction for RNA-Seq sequencing.

Table S5 Quality assessment of sample sequencing

Sample Name	Reads	Filtered Reads	Number of bases after filtering（bp）	Q20（%）	Q30（%）
A1	15,915,740	14,850,340	2,227,551,000	98.40	95.46
A2	16,416,620	15,333,034	2,299,955,100	98.31	95.32
A3	20,878,590	19,481,760	2,922,264,000	98.31	95.24
B1	18,407,480	17,285,748	2,592,862,200	98.24	94.91
B2	15,476,550	14,155,266	2,123,289,900	98.27	94.71
B3	16,490,912	15,209,394	2,281,409,100	98.18	94.54

Note: The constructed cDNA library was up-sequenced using the Illumina platform to obtain raw down-computed data, and the sequencing data were further filtered, and the sequencing data are shown in Table S5. The samples got 15,476,550~20,878,590 Raw Reads after offline, and 14,155,266~19,481,760 Clean Reads were obtained after cutting and removing the low-quality data, and the filtered base number was 2.12~2.92 Gb. The results showed that Q20% were all greater than 98%, and Q30% were all greater than 94%, indicating that the sequencing quality is high and can be used for subsequent analysis.
